# Downregulation of *Friend Leukemia Integration 1* (*FLI1*) follows the stepwise progression to gastric adenocarcinoma

**DOI:** 10.18632/oncotarget.26974

**Published:** 2019-06-11

**Authors:** Armando Del Portillo, Elena V. Komissarova, Aqiba Bokhari, Caitlin Hills, Anne Koehne de Gonzalez, Sarawut Kongkarnka, Helen E. Remotti, Jorge L. Sepulveda, Antonia R. Sepulveda

**Affiliations:** ^1^ Department of Pathology and Cell Biology, Columbia University Irving Medical Center, New York, NY, USA

**Keywords:** gastric cancer, FLI1, dysplasia, tumor suppressor, methylation

## Abstract

Gastric adenocarcinoma (GC) is a leading cause of cancer-related deaths worldwide. The transcription factor gene *Friend Leukemia Integration 1* (*FLI1*) is methylated and downregulated in human GC tissues. Using human GC samples, we determined which cells downregulate *FLI1*, when *FLI1* downregulation occurs, if *FLI1* downregulation correlates with clinical-pathologic characteristics, and whether *FLI1* plays a role in invasion and/or proliferation of cultured cells. We analyzed stomach tissues from 98 patients [8 normal mucosa, 8 intestinal metaplasia (IM), 7 dysplasia, 91 GC] by immunohistochemistry for FLI1. Epithelial cells from normal, IM, and low-grade dysplasia (LGD) showed strong nuclear FLI1 staining. GC epithelial cells showed significantly less nuclear FLI1 staining as compared to normal epithelium, IM and LGD (P=1.2×10^-5^, P=1.4×10^-6^ and P=0.006, respectively). *FLI1* expression did not correlate with tumor stage or differentiation, but was associated with patient survival, depending on tumor differentiation. We tested the functional role of FLI1 by assaying proliferation and invasion in cultured GC cells. Lentiviral-transduced *FLI1* overexpression in GC AGS cells inhibited invasion by 73.5% (P = 0.001) and proliferation by 31.5% (P = 0.002), as compared to controls. Our results support a combined role for FLI1 as a suppressor of invasiveness and proliferation in gastric adenocarcinoma, specifically in the transition from pre-cancer lesions and dysplasia to invasive adenocarcinoma, and suggest that FLI1 may be a prognostic biomarker of survival in gastric cancers.

## INTRODUCTION

Gastric cancer (GC) is the 5^th^ most common cancer and the 4^th^ leading cause of cancer-related deaths worldwide [1, 2]. The most common histopathologic type of stomach cancer is adenocarcinoma, also known as gastric adenocarcinoma [3]. Most gastric adenocarcinomas develop through a stepwise progression of histologic lesions, starting with gastritis, followed by intestinal metaplasia, dysplasia (low and high-grade), and eventually invasive adenocarcinoma [4, 5]. The main risk factor for gastric adenocarcinoma is *Helicobacter pylori*-associated chronic gastritis (reviewed in [6]). *Helicobacter pylori* infection of the stomach leads to interaction of the bacteria and inflammatory mediators with gastric epithelial cells, including progenitor and stem cells, resulting in accumulation of mutations, epigenetic modifications and deregulation of cellular function that may ultimately lead to dysplasia and adenocarcinoma [7, 8]. Along this cancer progression pathway, gastric epithelial cells undergo alterations in their transcriptional program, either due to genetic events (e.g. mutations, translocations, genetic losses and gains) or by epigenetic events, including DNA methylation.

DNA hypermethylation occurs in some subsets of gastric adenocarcinomas, namely Epstein-Barr virus (EBV)-associated gastric adenocarcinomas, gastric adenocarcinomas with microsatellite instability (MSI), and other gastric cancers with CpG island methylator phenotypes (CIMP) [9]. CpG methylation occurs early in gastric carcinogenesis, involving genes such as *MLH1* and *CDKN2A*, among others, and can be seen in non-neoplastic gastric epithelium as part of an epigenetic “field defect” [10–20]. Genome-wide methylation of gastric adenocarcinomas and gastric pre-cancer lesions have been characterized in a number of studies [9–21]. We recently reported the progressive downregulation of cancer-related genes by CpG methylation in gastric carcinogenesis, and found that *FLI1* gene hypermethylation was associated with reduced transcription in gastric adenocarcinoma tissues [21]. *FLI1* (Entrez Gene ID 2313) encodes an E26 transformation specific (ETS) family transcription factor that regulates genes involved in proliferation and differentiation, with previously reported roles mainly in endothelial and hematopoietic cells [22, 23]. The role of FLI1 in epithelial cells is not well known, and studies have shown conflicting roles for FLI1 in these cells. For example, two studies showed that overexpression of *FLI1* promotes malignancy in breast cancer [24, 25], while a different breast cancer model suggested that *FLI1* acts as a tumor suppressor gene [26]. Additionally, *FLI1* was shown to be commonly hypermethylated and downregulated in colorectal adenomas and adenocarcinomas [27, 28], further suggesting a possible tumor suppressor role in epithelial cells.

Since we previously found decreased transcription of *FLI1* in gastric adenocarcinoma tissues [21], we hypothesized that FLI1 may act as a tumor suppressor and that its expression would decrease in the progression of gastric carcinogenesis from normal epithelium to dysplasia and adenocarcinoma. However, previous studies examining hypermethylation and expression have only analyzed whole tissue, and thus the spatial and cellular distribution of FLI1 in gastric mucosa and in gastric adenocarcinoma cells have not been characterized. In order to examine which cells in gastric adenocarcinomas and pre-cancer lesions show altered *FLI1* expression, we analyzed formalin fixed paraffin embedded (FFPE) tissues by immunohistochemistry (IHC) for FLI1, and analyzed the expression pattern of *FLI1 in situ* in human stomach samples, including normal stomach antrum and body/fundus, intestinal metaplasia, dysplasia, and invasive adenocarcinoma. We then assessed potential associations of *FLI1* expression with tumor and clinical parameters, including tumor differentiation, stage, and survival. Further, we characterized the expression of *FLI1* in gastric cancer cell lines and determined the functional role of FLI1 in AGS gastric adenocarcinoma cells in culture by overexpression via lentiviral transduction.

## RESULTS

### Characterization of *FLI1* expression in normal, pre-neoplastic, and neoplastic gastric epithelial cells

To characterize the expression pattern of *FLI1* in gastric mucosa without intestinal metaplasia, mucosa with intestinal metaplasia (IM), dysplasia, and gastric adenocarcinoma, we stained and analyzed tumor microarrays (TMAs) and traditional whole tissue sections from 98 patients by H&E stains and IHC (see Table 1 for summary).

**Table 1:** Clinical and pathologic features of study cases

Parameter	Result
Gender	No. (%)
Male	61 (62%)
Female	37 (38%)
Age (years) range, median, mean (SEM)	16-95, 71, 69.6 (1.35)
Tumor stage (N = 91)	No. (%)
T1	9 (10%)
T2	14 (15%)
T3	34 (37%)
T4	34 (37%)
Histologic type (N = 91)	No. (%)
Intestinal	43 (47%)
Diffuse	20 (22%)
Mixed	19 (21%)
Other	5 (5%)
Not stated	4 (4%)
Differentiation (by report) (N = 91)	No. (%)
Well	5 (5%)
Moderate	17 (19%)
Moderate to poor	25 (3%)
Poor	41 (45%)
Undifferentiated component	2 (2%)
Not stated	1 (1%)
Tumor location (N = 91)	No. (%)
Antrum	37 (41%)
Body/fundus	18 (20%)
Cardia	5 (5%)
Gastroesophageal junction	15 (16%)
Cardia and fundus	5 (5%)
Broad involvement of stomach	9 (10%)
Unknown	2 (2%)
*H. pylori* status (N = 98)	No. (%)
Positive	25 (26%)
Negative	46 (47%)
Unknown	27 (28%)

Characteristics of all patients (N = 98) involved in this study (normal, IM, dysplasia, and adenocarcinoma) and of all adenocarcinomas in this study (N = 91). Abbreviations: No., number; SEM, standard error of the mean.

**Figure 1:**
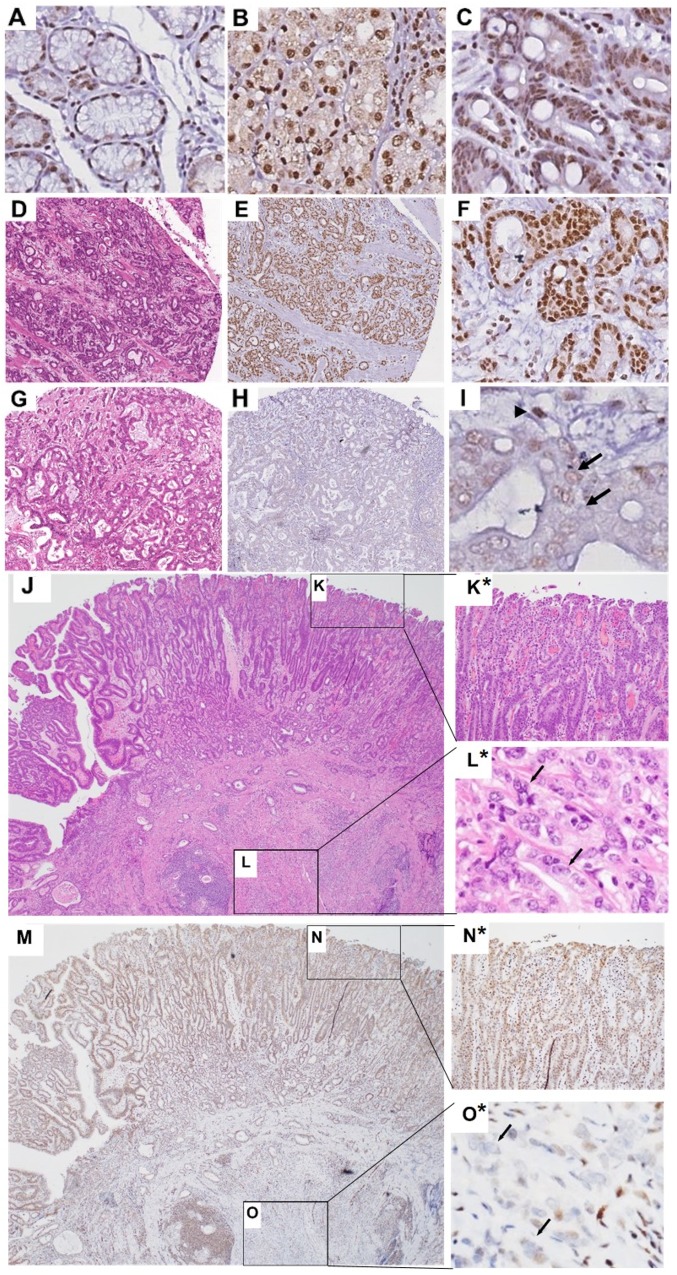
Representative nuclear FLI1 IHC staining in normal, IM and gastric adenocarcinoma tissues Antral glands **(A)**, oxyntic glands **(B)**, IM **(C)**, representative tumor with high FLI1 H-score of 199 **(D–F)**, representative tumor with low FLI1 H-score of 88 **(G–I)**. E, F, H and I show tumor samples with nuclear FLI1 IHC staining, at low (E and H) and high magnification (F and I). (I) Arrows point to tumor nuclei with little to no FLI1 positivity. Arrowhead points to stromal cell nucleus with strong FLI1 positivity, serving as internal positive control. A-D and G are H&E stains. Low power H&E **(J)** of stomach with high-grade dysplasia **(K)** and invasive adenocarcinoma **(L)**. High power view of K and L inset are shown as K^*^ and L^*^. Panel M shows FLI1 IHC of the case represented in panel J. High-grade dysplasia with preserved nuclear *FLI1* expression is shown in the inset N and as high power view in N^*^. Invasive adenocarcinoma with loss of nuclear *FLI1* expression is shown in the inset O and as high power view in O^*^, where the arrows indicate the nuclei of invasive adenocarcinoma. Original magnification 200×.

**Figure 2:**
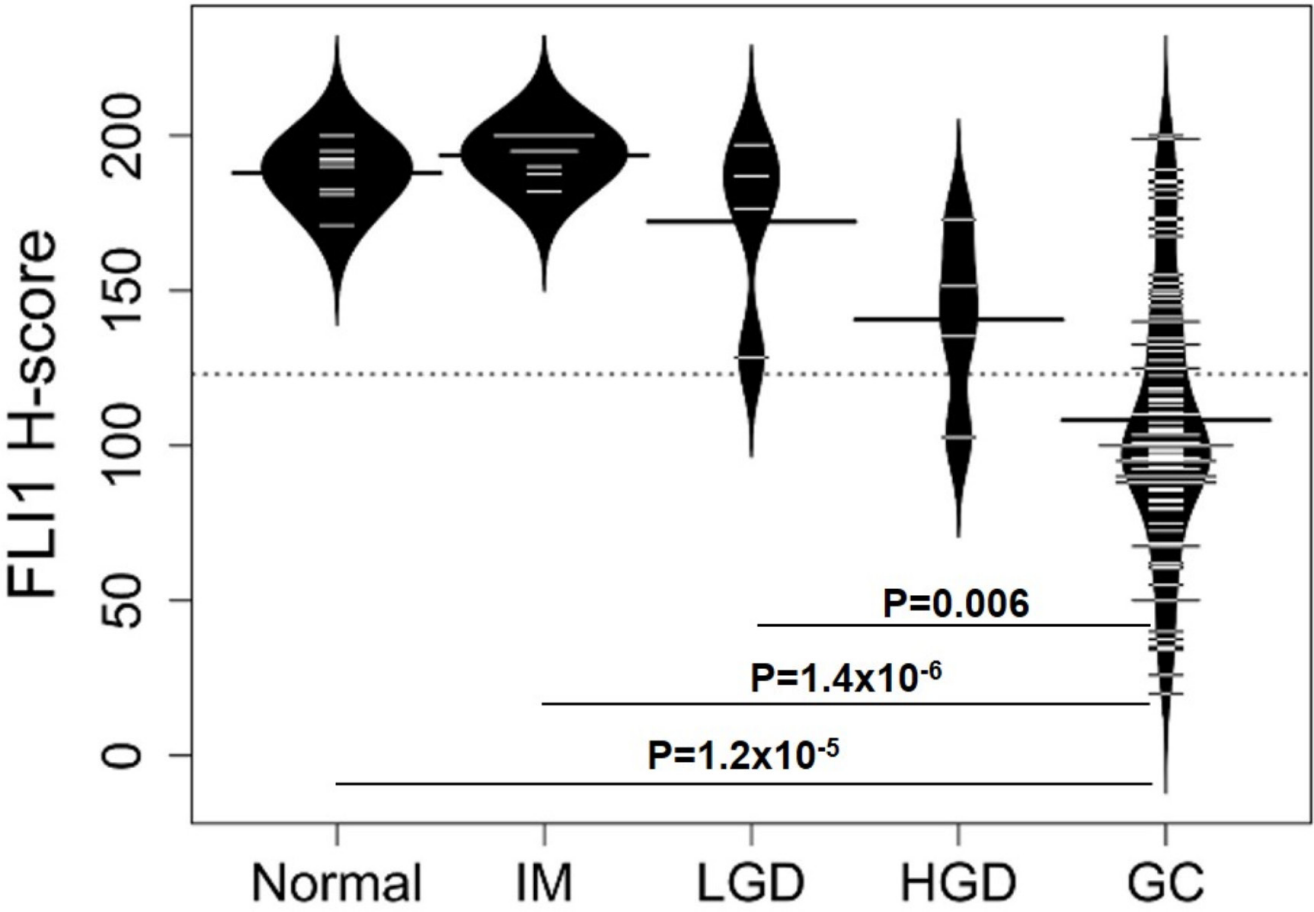
Distributions of FLI1 H-scores for different types of gastric tissue Bean plots showing the distribution of FLI1 H-scores for normal, intestinal metaplasia (IM), low-grade dysplasia (LGD), high-grade dysplasia (HGD), and gastric adenocarcinoma (GC), with mean H-scores (represented by the largest line in each bean plot) of 187.9 (SEM = 3.25), 193.7 (SEM = 2.37), 172.3 (SEM = 15.2), 140.6 (SEM = 14.8), and 108.2 (SEM = 14.6), respectively. A P value <0.05 was considered statistically significant. Significant differences in FLI1 H-scores were detected for normal vs GC (P = 1.2×10^-5^), IM vs GC (P = 1.4×10^-6^), and LGD vs GC (P = 0.006).

Since IHC for FLI1 showed variable staining in normal epithelium and gastric adenocarcinoma cells, we employed a composite scoring system taking into account both intensity and frequency of positive nuclei in the tissue of interest to generate an H-score [29], where the proportion and intensity scores only included epithelial cells. Additionally, for normal and intestinal metaplasia samples in the TMAs, the depth of gastric mucosa represented in the cores of tissue used to generate the TMAs varied, with some cores showing surface and deep glandular epithelium, and other cores showing just deep glandular epithelial cells. Thus, in order to compare scores fairly across tissue cores, we only evaluated deep glandular epithelial cells. Normal mucosa and IM samples were represented in their own cores in the TMAs, and thus were scored separately from tumor cores. We found strong nuclear FLI1 staining in endothelial and hematopoietic cells in the lamina propria, as expected, which served as internal positive controls. Normal gastric glandular epithelial cells showed strong nuclear FLI1 staining in deep antral and oxyntic glands, with mean H-score of 187.9 (Figures 1A and 1B). Epithelial cells of deeper areas of intestinal metaplasia also showed strong nuclear staining with mean H-score of 193.7 (Figure 1C). Normal antral mucous gland epithelial cells and oxyntic epithelial cells showed similar nuclear FLI1 expression (Figure 1A and 1B, respectively). Furthermore, intestinal metaplasia epithelial cells showed strong FLI1 expression, which was not significantly different from normal antral/oxyntic epithelium (Figure 1C and Figure 2).

The distributions and mean H-scores for epithelial cells of normal mucosa, intestinal metaplasia, low- and high-grade dysplasia and adenocarcinoma are shown in Figure 2. The mean H-scores did not show a statistically significant difference between low-grade (172.3) and high-grade dysplasia (140.6, P = 0.22, Dunn test). Low-grade dysplasia and high-grade dysplasia did not significantly differ from normal stomach glands (P = 0.33 and 0.09, respectively). Gastric adenocarcinomas showed variable nuclear *FLI1* expression (Figures 1D–1I) in the malignant epithelial cells, with overall downregulation and H-scores ranging from a high of 200 to a low of 20. However, most gastric adenocarcinomas showed weak nuclear FLI1 staining with an overall mean H-score of 108.2, which was significantly lower than normal stomach glands (P = 1.2×10^-5^), intestinal metaplasia (P = 1.4×10^-6^), and low-grade dysplasia (P=0.006) (Figure 2).

One patient’s endoscopic mucosal resection specimen showed areas of both high-grade dysplasia and adenocarcinoma (Figure 1J–1O). In this patient, the high-grade dysplastic epithelial cells showed strong nuclear *FLI1* expression (Figure 1N^*^, mean H-score = 151.5), but the deepest invasive tumor epithelial cells showed almost absent nuclear signal (Figure 1O^*^, mean H-score = 35), suggestive of a stepwise downregulation of *FLI1*. Gastric adenocarcinomas in the TMAs showed variable levels of nuclear *FLI1* expression, but 96% of these tumors had an H-score less than the mean score for normal gastric glands. Overall, the distribution of H-scores for each tissue type shows a pattern of progressively lower nuclear *FLI1* expression from non-dysplastic tissues of normal mucosa and intestinal metaplasia to dysplasia, to cancer, reaching a statistically significant drop in expression in adenocarcinoma epithelial cells (Figure 2).

### Correlation between *FLI1* expression and tumor differentiation, tumor stage, and patient survival

We then asked if FLI1 could serve as a marker for prognosis by determining if FLI1 IHC H-scores correlated with tumor differentiation, tumor stage (T1 to T4) or patient survival. The highest stage of either the pathologic T stage at the time of resection or the clinical T stage at the time of biopsy was considered for each case. There were no significant differences in FLI1 nuclear H-scores between pathologic/clinical T stages or tumor differentiation (P>0.05, Dunn test).

**Figure 3:**
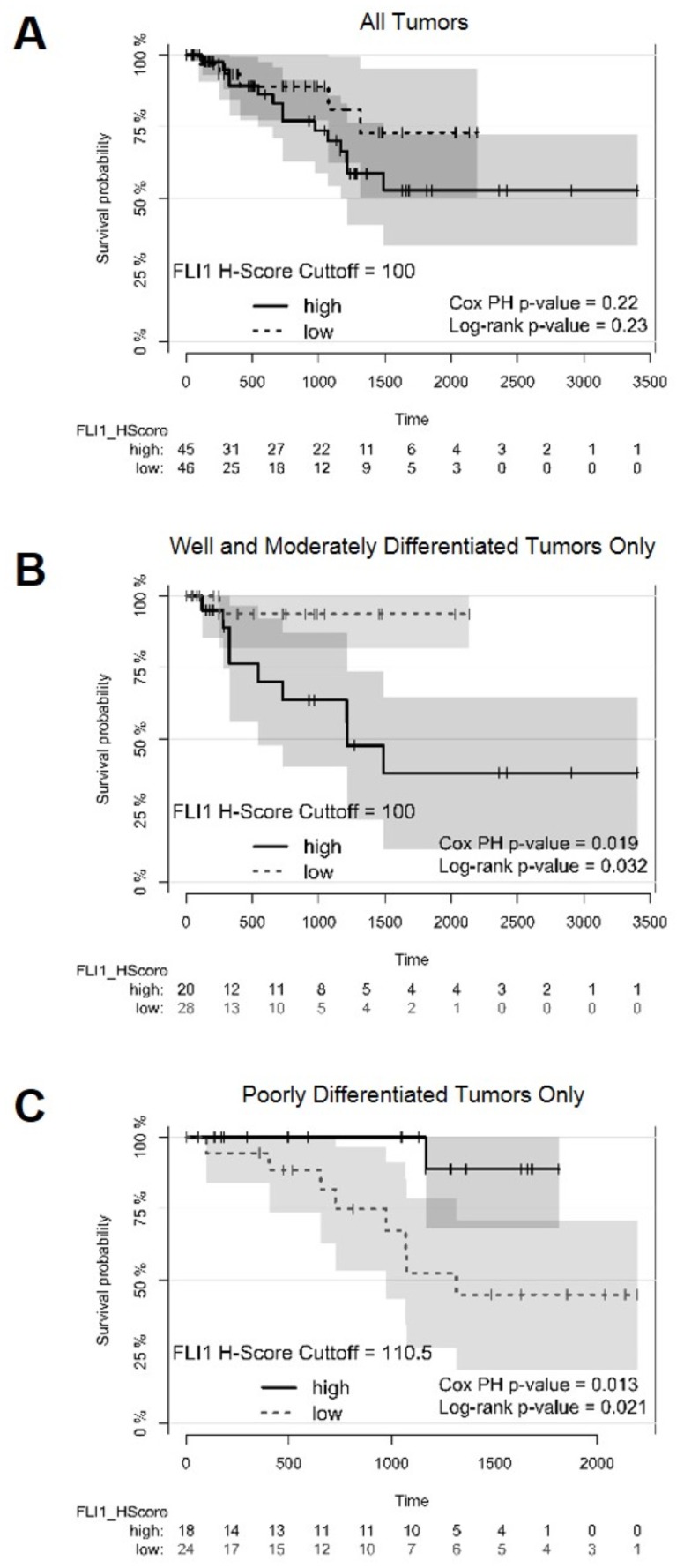
Survival plots of patients with optimal cutoffs for high and low FLI1 H-scores Survival plots displaying survival probability (y-axis) vs time in days (x-axis). **(A)** Survival plot for all patients analyzed in the TMAs with FLI1 H-score cutoff of 100 to separate high and low *FLI1*-expressing tumors. **(B)** Survival plot for all patients with well and moderately differentiated tumors, using a FLI1 H-score cutoff of 100 to separate high and low *FLI1*-expressing tumors. **(C)** Survival plot for all patients with poorly differentiated tumors, using a FLI1 H-score cutoff of 110.5 to separate high and low *FLI1*-expressing tumors. The Cox proportional hazards (PH) model and the Mantel-Haenszel log-rank P values are displayed. A P value <0.05 was considered statistically significant.

The overall survival probability for all tumors (stages 1 to 4 and including well, moderately and poorly differentiated tumors together), did not show differences between tumors with a high FLI1 H-score (100 or greater) and a low FLI1 H-score (<100) (Figure 3A). However, when tumors that were well and moderately differentiated or poorly differentiated were analyzed separately, the high *FLI1*-expressing well and moderately differentiated tumors had worse survival than those with low *FLI1* expression (Figure 3B). Intriguingly, the high *FLI1*-expressing poorly differentiated tumors had improved survival as compared to those with low *FLI1* expression (Figure 3C), suggesting a role for FLI1 as a differentiation-dependent tumor prognostic biomarker.

### Overexpression of *FLI1* inhibits proliferation and invasion in the AGS human gastric adenocarcinoma cell line

**Figure 4:**
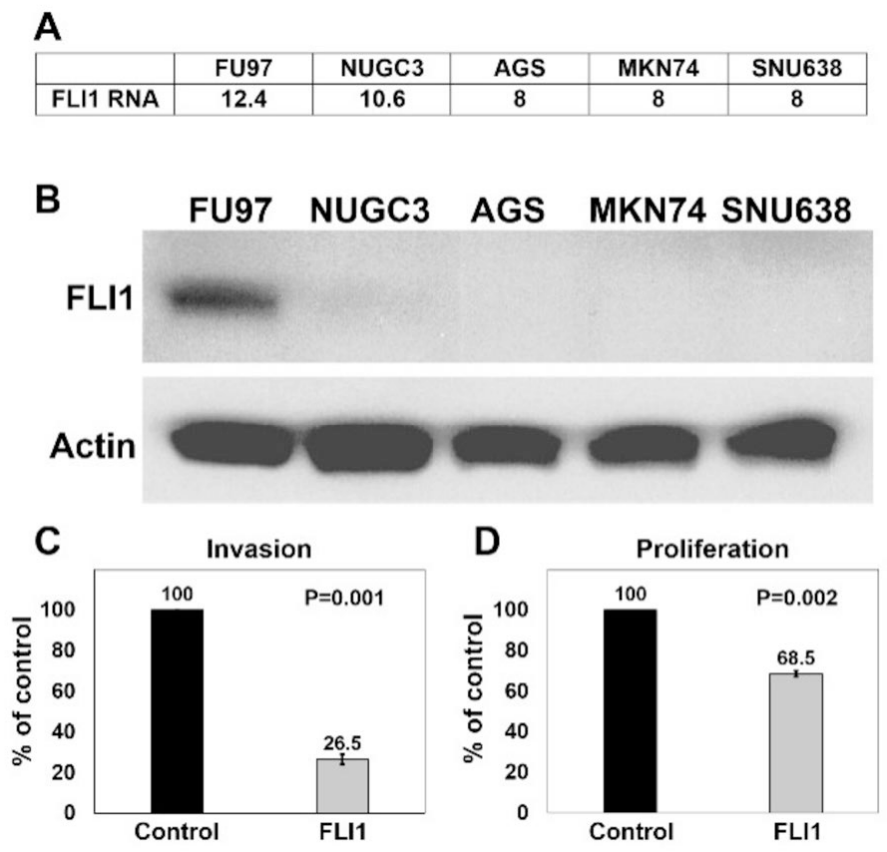
Overexpression of *FLI1* in AGS cells inhibits invasion and proliferation **(A)** Normalized (log2) expression (Fragments per Kb of transcript per Million fragments mapped, FPKM) of *FLI1* in gastric adenocarcinoma cell lines (FU97, NUGC3, AGS, MKN74, SNU638). **(B)** Western blots performed with anti-FLI1 (top, ~55kDa) and anti-β-actin (bottom, ~42kDa) antibodies on protein extracts from human gastric adenocarcinoma cell lines. Invasion assay **(C)** and proliferation assay **(D)** of AGS cells transduced with either control or *FLI1* expression vector lentiviral particles.

We asked if human gastric cancer cell lines also show similar reduced levels of *FLI1* expression as compared to adenocarcinoma cells *in vivo*. First, we interrogated a transcriptional database derived from RNA-Seq data from human gastric cancer cell lines [30], and found that most of these cell lines do not express *FLI1* (Figure 4A). The highest *FLI1*-expressing gastric cancer cell line, FU97, is an unusual variant of gastric cancer, showing alpha-fetoprotein production [31]. Other commonly used gastric adenocarcinoma cell lines, such as AGS, showed no detectable *FLI1* transcription. To confirm these findings, we performed a western blot (Figure 4B) on two gastric adenocarcinoma cell lines that expressed *FLI1* RNA (FU97 and NUGC3) and on three gastric adenocarcinoma cell lines that did not (AGS, MKN74, and SNU638). The protein levels by western blot correlated with the RNA transcription data, where FU97 showed the most FLI1 protein production, NUGC3 showed little FLI1 protein, and the other cell lines showed no detectable FLI1 protein.

Our results show that FLI1 is commonly lost in gastric adenocarcinomas, but methylation of *FLI1* promoter and loss of expression may be a surrogate marker for global methylation rather than having a functional role in gastric tumorigenesis. To assess if FLI1 has a functional role in gastric adenocarcinoma, we overexpressed either control plasmid or *FLI1* in AGS cells by lentiviral transduction, and determined the effect of *FLI1* expression on invasion and proliferation (Figures 4C and 4D). Similar proportions of AGS cell transduction were seen between control and *FLI1* lentiviral particles. *FLI1* overexpression was confirmed by GFP expression using fluorescence microscopy and by quantitative RT-PCR from three independent experiments, which ranged from 49 to 48,722 fold expression as compared to control lentiviral transduced cells (mean = 16,341, SEM = 16,190). Using a Matrigel invasion assay, overexpression of *FLI1* reduced AGS cell invasion by 73.5% as compared to control cells (Figure 4C, P = 0.001). In parallel, a proliferation assay showed that overexpression of *FLI1* reduced proliferation of AGS cells by 31.5% (Figure 4D, P = 0.002). These results support an invasive suppressor role for FLI1 in gastric adenocarcinoma.

## DISCUSSION

We previously reported that there was decreased *FLI1* RNA in gastric adenocarcinoma tissues as compared to intestinal metaplasia, which inversely correlated with *FLI1* promoter methylation levels, but the cellular localization of FLI1 in cancer tissues has not been determined [21]. In the current study, we report that nuclear *FLI1* expression decreases along the progression to adenocarcinoma, with preserved nuclear expression in normal antral and oxyntic glands and intestinal metaplasia, and decreased expression from low-grade dysplasia and high-grade dysplasia to the lowest expression in invasive gastric adenocarcinomas.

We then analyzed how nuclear *FLI1* expression correlated with tumor and clinical features. Loss of nuclear *FLI1* expression did not correlate with tumor stage or tumor differentiation. However, patients with low FLI1 H-scores in well and moderately differentiated adenocarcinomas survived longer than patients with high FLI1 H-scores. Interestingly, the opposite observation was seen in patients with poorly differentiated adenocarcinomas. That is, for patients with poorly differentiated tumors, patients with low FLI1 H-scores had worse survival. One possible explanation for this observation is that FLI1 may play alternative roles and differentially affect survival in various gastric cancer sub-types characterized by different molecular pathways, histological differentiation and/or tumor microenvironment.

We also tested if gastric adenocarcinoma cell lines show loss of *FLI1* expression, similar to our *in vivo* observations. By RNA analysis, only rare gastric adenocarcinoma cell lines showed *FLI1* expression, with FU97 showing the highest level of expression. Western blot results correlated with transcriptional data, confirming high expression of *FLI1* in FU97, low *FLI1* expression in NUGC3, and the lack of *FLI1* expression in more commonly used cell lines such as AGS, MKN74 and SNU638. FU97 is a gastric adenocarcinoma cell line that also expresses alpha-fetoprotein, which represents a rare variant of gastric adenocarcinoma [31]. Thus, the significance of high *FLI1* expression in this unusual cell line is unclear. However, the majority of gastric adenocarcinoma cell lines do not express *FLI1*, which is in agreement with our results from IHC on primary human samples. Additionally, we showed evidence that FLI1 has a functional role in gastric adenocarcinoma, rather than acting as a surrogate marker for global DNA methylation. AGS cells overexpressing *FLI1* from a lentiviral vector showed reduced levels of invasion and proliferation as compared to control AGS cells. Since invasion was assayed by quantifying the number of cells that invaded through a Matrigel-coated membrane, the reduction of proliferation in AGS cells overexpressing *FLI1* would affect the invasive assay quantification. However, since the reduction in proliferation was much smaller than the level of reduction of invasion (31.5% vs 73.5%), we conclude that the reduction in invasion was at least partly due to a change in the invasive properties of the tumor cells. Therefore, our findings suggest a functional role of FLI1 in suppressing invasion of gastric cancer cells. Further, our finding that FLI1 affects invasiveness and proliferation in the AGS cell line warrants future studies to detail whether motility is affected in conjunction with or independent of cell proliferation and viability. Future studies to characterize the mechanisms of FLI1 regulation and the role of FLI1 in additional cell lines as well as in animal models are warranted.

FLI1 is best known for its role in Ewing’s sarcoma where a translocation event between chromosomes 11 and 22 results in an oncogenic EWS-FLI1 fusion protein. This fusion protein aberrantly activates or represses enhancer elements via the N-terminal transactivation domain from EWS and the C-terminal DNA binding domain of FLI1 [32]. However, this oncogenic potential is dependent on the chimeric nature of the fusion protein, and does not necessarily reflect the native function of FLI1. Knockout of *Fli1* in mice is embryonic lethal, and these mice show vascular abnormalities and thrombocytopenia, pointing to an essential role of *FLI1* in endothelial cells and megakaryocytes [33]. In humans, Paris-Trousseau syndrome demonstrates the native role of *FLI1*, whereby the loss of chromosome 11q, the location of *FLI1*, leads to thrombocytopenia following defects in megakaryocytes [33, 34]. While there is a clear role for FLI1 in hematopoietic cells, in particular megakaryocytes, its role in epithelial cells has not been fully elucidated. The tissues with the highest *FLI1* expression are spleen, lymph node, appendix, placenta, and lung, [35] all of which are rich in hematopoietic and/or endothelial cells. However, few studies examined the functional role for FLI1 in epithelial cells. In breast cancer studies, there are conflicting results regarding the role of FLI1 in these cells. In a murine breast cancer model, Fli1 was shown to act as a tumor suppressor [26], but studies examining human breast cancer showed that FLI1 enhances tumor properties [24, 25] and that its expression correlates with poor prognostic factors such as lymph node metastasis and poor differentiation [25]. In oral squamous cell carcinoma (OSCC), FLI1 served as a prediction marker for radiotherapy resistance [36]. In that study, *FLI1* expression was detected in epithelial cells by IHC but the staining pattern was cytoplasmic, not nuclear, the significance of which is uncertain given that it is known to function as a transcription factor. Interestingly, however, as compared to normal tissue, the *FLI1* gene was methylated in OSCCs [37]. *FLI1* is also downregulated in intraductal papillary mucinous neoplasms (IPMNs) [38] and in colorectal adenocarcinomas [27, 28]. In a comprehensive TMA study evaluating many different types of tumors by IHC, only 3% of stomach cancers (2/67) showed FLI1 positive staining [39], supporting our findings that FLI1 is commonly lost in gastric adenocarcinomas. While FLI1 has a known role in hematopoiesis, these studies lend evidence towards a tumor suppressor role for FLI1 in various epithelial tumors, especially in the gastrointestinal tract.

In our previous study where we analyzed the TCGA gastric adenocarcinoma dataset [21], we showed that *FLI1* was methylated in gastric adenocarcinomas, and its expression was inversely correlated with its level of methylation. Moreover, *FLI1* expression was lowest in microsatellite-unstable (MSI) tumors compared with other gastric cancer molecular subtypes.

The clinical GC samples in the TMAs used in our current study were not classified as EBV (Epstein Barr virus), MSI (microsatellite instability), CIN (chromosomal instability), or GS (genomically stable) subtypes [9]. However, we performed EBV *in situ* hybridization and only 4 GC cases were EBV positive. We also performed MLH1 immunostaining of our TMAs and detected only 8 GC cases with loss of MLH1 indicating these tumors were of MSI type (data not shown). The small numbers of MLH1 deficient/MSI cases in our study is insufficient to assess the association of MSI and FLI1. However, these data show that most of our cases were of the CIN or GS subtypes.

In summary, our data show that *FLI1* expression is commonly downregulated in gastric cancers, support a combined role for FLI1 as a suppressor of invasiveness and proliferation in gastric cancer, in particular in the transition from pre-cancer lesions (IM and dysplasia) to invasive adenocarcinoma, and suggest that FLI1 may be a prognostic biomarker of survival in specific sub-groups of gastric cancers.

## MATERIALS AND METHODS

### Tissue samples and construction of gastric adenocarcinoma microarray

This study was approved by an institutional review board at Columbia University. A search in our pathology database of the Department of Pathology and Cell Biology, Columbia University, was performed to identify surgical resection specimens for gastric and gastroesophageal junction adenocarcinomas between 2002 and 2012. Slides from formalin fixed, paraffin embedded (FFPE) tissue blocks were reviewed to select areas of tumor, normal stomach mucosa and intestinal metaplasia to generate six tumor microarrays (TMAs), representing 110 cases of gastric adenocarcinoma. The cores in the microarray were 2 mm in diameter, and 54 cores were placed on each TMA, with two additional cores of control placental tissue. For each tumor case, there were at least two cores, from representative cancer areas. When selecting for normal gastric mucosa and IM, we selected areas away from the tumor, such that each core was usually dedicated to a single tissue type, allowing a separate scoring of malignant and non-malignant cells. Further, in TMA cores containing cancer tissue, only malignant epithelial cells were scored for FLI1, while non-malignant epithelial cells or any other cells present were not scored.

Additionally, we searched our pathology database for cases of dysplasia in the stomach between 7/2016 to 5/2017, which yielded seven cases, including one case showing low-grade dysplasia and high-grade dysplasia, and one case showing high-grade dysplasia and gastric adenocarcinoma. Of these patients, three had a history or a concurrent diagnosis of gastric adenocarcinoma, and one developed an adeno-squamous carcinoma of unknown origin. The other three patients with dysplasia had no history or development of gastric adenocarcinoma, per our clinical records. The sections of dysplasia and the TMAs were stained with hematoxylin and eosin (H&E) for histological assessment of morphology, grade of dysplasia, and tumor grade.

### FLI1 immunohistochemistry (IHC) and scoring

Tissue sections were stained using a clinically validated mouse monoclonal antibody to human FLI1 (clone MRQ-1, Cell Marque). We used a clinically validated automated immunostaining protocol for FLI1 that is routinely used for clinical samples in an automated Leica Bond immunostaining system, and staining of our research samples were performed alongside clinical samples, using the clinical control tissues (skin, tonsil, bowel and spleen).

Briefly, slides of FFPE 5 micron thick tissue sections were incubated in Leica Bond ER2 solution (pH 9) for 20 minutes, and then labeled with Bond Polymer Refine Detection along with anti-FLI1 antibody for 30 minutes. FLI1 IHC slides were scored on a scale of 0, 1, or 2, based on nuclear intensity, where a score of 0 was given for no nuclear signal, a score of 1 for mild to moderate nuclear signal, and a score of 2 for strong nuclear signal similar to the intensity seen in internal positive controls (endothelial cells and lymphocytes). Cytoplasmic staining when present was minimal, and was interpreted to represent background stain that was not scored. In order for a core to be considered adequate for scoring, clear tissue of interest (normal, IM, or gastric adenocarcinoma) must have been present and recognizable to the pathologist. Additionally, at least 50 cells of interest needed to be present. If there were too few cells or if it was too difficult to distinguish poorly differentiated cancer cells from background stromal cells, the core was not scored.

To capture the variability within a single sample, we also estimated the percentage of cells of interest (antral or oxyntic glands, intestinal metaplasia, or neoplastic cells) for each score. An H-score [29] was then calculated by multiplying the percentage (x 100) of cells with a given score by the score value, and taking the sum, for a maximum score of 200. For normal gastric mucosa and intestinal metaplasia tissues, the deeper glandular epithelial cells were scored because surface epithelium was not always present in TMAs. The 2 mm cores in the TMAs were generally of sufficient diameter to contain enough architecture to evaluate normal mucosa and IM, and deeper glandular areas were easily identified in some TMA cores that had adequate orientation of the mucosa. In other TMA cores, the surface of the mucosa was not completely represented, since we could not identify the surface epithelium, but all cases had deep glandular profiles, which we could easily identify by their proximity to the muscularis mucosae and/or the typical histologic features of gastric glands.

To minimize potential scoring bias, we scored the TMAs cores in a blinded fashion, and consecutive scoring of duplicates from the same patient was systematically avoided. Once the H-scores were calculated, the key to the TMAs was revealed to determine if two or more samples came from the same tumor, and mean scores were calculated.

Of the 110 cases of gastric adenocarcinoma in the TMA, 91 had adequate tissue for scoring. Eighty-four tumor samples had at least two cores represented in the TMAs and an average of the H-scores was taken for these cases. Seven tumor samples had only one core represented in the TMAs. One specimen had two simultaneous primary gastric adenocarcinomas that were staged as pT3 and pT1, but only the pT3 lesion was sampled for the TMA. Nineteen tumor cases were not scored due to difficulty in distinguishing tumor cells from normal cells or due to the lack of tumor cells (e.g. due to tissue loss or non-representative portion of tumor in the TMA). There were eight patients with normal stomach controls and eight with intestinal metaplasia, and six patients in each of these groups had two cores of tissues scored in the TMAs. In addition to the cases represented in the TMAs, we also analyzed seven patients with dysplasia. Among the seven patients with dysplasia, three had a history or a concurrent diagnosis of gastric adenocarcinoma, and one developed an adeno-squamous carcinoma of unknown origin. The other three patients with dysplasia had no history of gastric adenocarcinoma. Staining of cases with dysplasia was performed using whole sections of tissue, and lesions were scored by taking the mean of two representative areas of interest.

### Tumor differentiation and staging

Tumor staging and differentiation information was collected from pathology reports. If a patient received treatment prior to resection, the higher T stage of either clinical-radiologic or pathologic staging was recorded. All tumors were staged according to the American Joint committee on Cancer (AJCC) 7^th^ edition. If a tumor was characterized as “moderately to poorly differentiated” or “moderately to focally poorly differentiated,” then the tumor was classified as moderately differentiated for this study.

### Statistical analyses

Since the FLI1 H-score data failed all normality tests (R package “nortest” version 1.0-4) the non-paired Wilcoxon rank sum test with continuity correction was used to compare FLI1 H-scores between two groups. The non-parametric Kruskal-Wallis rank sum test with the post-hoc Dunn test for multiple comparisons was used to compare differences between expression of *FLI1* in multiple groups. The Fisher’s exact test was performed to evaluate the frequency of tumor stage or tumor differentiation phenotypes in low or high *FLI1*-expressing gastric adenocarcinomas. Kaplan-Meier plots were performed using the “prodlim” plot in R after separating the H-scores into high and low scores based on determination of the optimal cutpoint using the maximally selected rank statistic from the ’maxstat’ R package, as implemented in the ‘survminer’ package. The optimally dichotomized FLI1 H-score was also used as a variable in univariate Cox proportional hazard model using the “coxph” function in the ‘survival’ package. For all statistics, a P value less than 0.05 was considered significant.

### Cell lines and western blot

The gastric adenocarcinoma cell lines FU97 and NUGC3 were obtained from the Japanese Collection of Research Bioresources Cell Bank via Xenotech (Lenexa, KS). The gastric adenocarcinoma cell lines AGS, MKN74 and SNU638 were obtained from American Type Culture Collection (ATCC). Cell lines were grown in Glutagro RPMI 1640 medium Corning Cellgro, except FU97 in DMEM with L-glutamine and sodium pyruvate Corning Cellgro (Mediatech, Inc. A Corning Subsidiary, Manassas, VA, USA), supplemented with 10% FBS Gibco/BRL (Life Technologies Corp., Grand Island, NY, USA) at 37°C in a 5% CO_2_ humidified atmosphere. Cell lines were generally used the 2^nd^ passage after thawing for experiments.

For western blots, cell lysates containing equal protein amounts were separated by SDS-PAGE electrophoresis and transferred to Immobilon-P PVDF membranes (Millipore Corp., Billerica, MA, USA). Membranes were probed with a rabbit polyclonal FLI1 antibody, catalog # PA5-29597 (Thermo Fisher Scientific, Rockford, IL, USA) followed by horseradish peroxidase-conjugated goat anti-rabbit antibody (Pierce, Rockford, IL, USA). The housekeeping gene β-actin was detected with mouse monoclonal HRP-conjugated antibody C4 (Santa Cruz Biotechnology, Inc., Dallas, TX, USA). Protein bands were detected using Pierce ECL 2 Western Blotting Kit (Thermo Fisher Scientific, Rockford, IL, USA), followed by exposure on Blue Light Autoradiography film (Fisher Scientific, Pittsburgh, PA, USA). Western blot films were scanned using a DocuMate 3115 scanner (Xerox, Rockleigh, NJ, USA) and image processing and analysis were performed using ImageJ.

### Real-time quantitative RT-PCR (qPCR)

To produce cDNA we used the Cells-to-cDNA II procedure. Briefly, transduced cells were washed in PBS and then heated in Cell Lysis II buffer (Thermo Fisher Scientific, Rockford, IL, USA) following the manufacturer protocol. Next, the crude lysates were treated with DNase I (Thermo Fisher Scientific, Rockford, IL, USA), and after DNase I inactivation, used for cDNA synthesis with qScript Flex cDNA Synthesis Kit (Quanta Biosciences, Beverly, MA) following the manufacturer protocol. The first strand cDNA was used as a template in qPCR. Pre-designed KiCqStart Primers for human *FLI1* (NM_002017.4) were purchased from Sigma-Aldrich (St. Louis, MO, USA). Real-time qPCR was carried out in technical duplicates using Power SYBR Green PCR Master Mix (Applied Biosystems, Carlsbad, CA, USA) and 7300 Real Time PCR System (Applied Biosystems, Carlsbad, CA, USA). qPCR data for each gene were normalized to the *GAPDH* housekeeping gene expression level in the sample. Relative quantification of the gene expression was performed with the comparative ^ΔΔ^C_T_ method.

### Lentiviral transduction

Lentiviral particles containing human *FLI1* (NM_002017.4) ORF under CMV promoter (LPP Z7405-Lv205-200) and empty control lentiviral particles (EX-NEG-Lv205) both containing GFP reporter were purchased from GeneCopoeia, Inc. (Rockville, MD, USA). Viral transductions of cells were performed following the manufacturer protocol. Briefly, 2×10^4^ cells per well were plated into the 12-well plate the day before transduction. Lentiviral particles (~80 MOI) were added to cell cultures in the presence of 6 ug/ml of polybrene. The expression of GFP was evaluated with a fluorescent microscope 4 days post-transduction.

Since each experiment infected only 2 × 10^4^ AGS cells with lentiviral particles in order to achieve a high enough infection frequency to perform experiments, we had just enough cells to perform invasion, proliferation, and qPCR assays from the same viral transduction well. Therefore, we chose to confirm that the transduced cells indeed expressed FLI1 mRNA levels rather than determine the protein levels since we would not be able to obtain sufficient protein for western blots. However, in early experiments, we had transduced NUGC3 cells and western blot results showed that the viral construct resulted in overexpression of FLI1 as compared to control vector (data not shown). We did not pursue the proliferation and invasion assays with NUGC3 cells because they normally express some, albeit low levels of FLI1, whereas AGS cells do not express detectable FLI1. Nonetheless, FLI1 expression driven by the FLI1 lentivirus, can be assessed by GFP expression in the transduced cells, since the lentiviral plasmid contains the *FLI1* gene and a GFP reporter expressed from an internal ribosomal entry site, thereby ensuring a 1:1 expression ratio of *FLI1* and GFP.

### Cell invasion assay

Cell invasion assays were performed with the Corning BioCoat Tumor Invasion System 96-Multiwell Format (Discovery Labware, Inc., Bedford, MA, USA) following the manufacturer protocol. Briefly, four days after transduction with lentiviral particles, cells were evaluated under fluorescent microscope and GFP expressing cells were counted for transduction efficiency evaluation. Then, the cells were pre-labeled with 10ug/ml DilC_12_(3) Fluorescent Dye (Discovery Labware, Inc. Bedford, MA) in growth medium for 1hr at 37°C. Cell suspensions were prepared by trypsinizing the monolayer and re-suspending in RPMI 1640 medium without FBS. Cell suspensions with the same transduction efficiencies were used in the assay. The Corning BioCoat 96-well plate was prepared by rehydrating the Matrigel matrix coating according to the manufacturer protocol, and cell suspensions (1.25 × 10^4^ cell/well) were added to the apical chambers. Chemoattractant RPMI 1640 supplemented with 10% FBS was added to each basal chamber. Following 48hr incubation at 37°C in a 5% CO_2_ humidified atmosphere, fluorescence reading was performed and data collected using the EnVision Parker Elmer Plate Reader at excitation/emission 549/565nm for DilC_12_(3). Only those fluorescent cells that passed through the Matrigel matrix layer and the membrane were detected. The assay was performed in triplicate. Data were analyzed and expressed as percent of control cells transduced with non-targeting lentiviral particles and background was subtracted prior to the calculation.

### Cell proliferation assay

Cell proliferation assays were performed in triplicate with CellTiter 96 Aqueous One Solution Reagent (Promega, Madison, WI, USA) according to the manufacturer recommendations. Briefly, four days after transduction, the cell suspensions with the same transduction efficiency were plated (5 × 10^3^ cells/well) in a 96-well plate. After 48 hrs, CellTiter 96 Aqueous One Solution Reagent was added to each well. Following 2 hr incubation at 37°C in a 5% CO_2_ humidified atmosphere, absorbance reading was performed using the EnVision Parker Elmer Plate Reader at 490nm. Data were analyzed and cell viability was expressed as percent of viable control cells transduced with control lentiviral particles and background was subtracted prior to the calculation.

## Abbreviations

FLI1: Friend Leukemia Integration 1
IHC: immunohistochemistry
H. pylori: Helicobacter pylori
EBV: Epstein-Barr Virus
MSI: microsatellite instability
CIMP: CpG island methylator phenotype
ETS: E26 transformation specific
FFPE: formalin-fixed, paraffin embedded
TMA: tissue microarray
LGD: low-grade dysplasia
HGD: high-grade dysplasia
GC: gastric adenocarcinoma
AJCC: American Joint Committee on Cancer
OSCC: oral squamous cell carcinoma
IPMN: intraductal pancreatic mucinous neoplasm
SEM: standard error of mean

## References

[1] Ferlay J, Soerjomataram I, Ervik M, Dikshit R, Eser S, Mathers C, et al. GLOBOCAN 2012 v1.0, Cancer Incidence and Mortality Worldwide: IARC Cancer Base No. 11 [Internet]. Lyon, France: International Agency for Research on Cancer; 2013 Available from: http://globocan.iarc.fr. Accessed on 12/10/2017.

[2] World Health Organization. Cancer Fact Sheets. Available from http://www.who.int/mediacentre/factsheets/fs297/en/. Accessed 13/10/2017.

[3] Gastric Cancer Treatment. (PDQ^®^)–Health Professional Version as was originally published by the National Cancer Institute, available at https://www.cancer.gov/types/stomach/hp/stomach-treatment-pdq. Accessed on 2/12/18.

[4] Correa P. Gastric cancer: overview. Gastroenterol Clin North Am. 2013; 42:211–17. 10.1016/j.gtc.2013.01.002. 23639637 PMC3995345

[5] Correa P, Piazuelo MB. The gastric precancerous cascade. J Dig Dis. 2012; 13:2–9. 10.1111/j.1751-2980.2011.00550.x. 22188910 PMC3404600

[6] Herrero R, Parsonnet J, Greenberg ER. Prevention of gastric cancer. JAMA. 2014; 312:1197–98. 10.1001/jama.2014.10498. 25247512

[7] Sepulveda AR. Helicobacter, Inflammation, and Gastric Cancer. Curr Pathobiol Rep. 2013; 1:9–18. 10.1007/s40139-013-0009-8. 23687623 PMC3655720

[8] Uehara T, Ma D, Yao Y, Lynch JP, Morales K, Ziober A, Feldman M, Ota H, Sepulveda AR. H. pylori infection is associated with DNA damage of Lgr5-positive epithelial stem cells in the stomach of patients with gastric cancer. Dig Dis Sci. 2013; 58:140–49. 10.1007/s10620-012-2360-8. 22945475 PMC3816997

[9] Cancer Genome Atlas Research Network. Comprehensive molecular characterization of gastric adenocarcinoma. Nature. 2014; 513:202–09. 10.1038/nature13480. 25079317 PMC4170219

[10] Maekita T, Nakazawa K, Mihara M, Nakajima T, Yanaoka K, Iguchi M, Arii K, Kaneda A, Tsukamoto T, Tatematsu M, Tamura G, Saito D, Sugimura T, et al. High levels of aberrant DNA methylation in Helicobacter pylori-infected gastric mucosae and its possible association with gastric cancer risk. Clin Cancer Res. 2006; 12:989-95. 10.1158/1078-0432.CCR-05-2096. 16467114

[11] Nakajima T, Maekita T, Oda I, Gotoda T, Yamamoto S, Umemura S, Ichinose M, Sugimura T, Ushijima T, Saito D. Higher methylation levels in gastric mucosae significantly correlate with higher risk of gastric cancers. Cancer Epidemiol Biomarkers Prev. 2006; 15:2317–21. 10.1158/1055-9965.EPI-06-0436. 17119066

[12] Tahara T, Arisawa T. DNA methylation as a molecular biomarker in gastric cancer. Epigenomics. 2015; 7:475–86. 10.2217/epi.15.4. 26077432

[13] Tahara T, Arisawa T, Shibata T, Nakamura M, Yoshioka D, Okubo M, Maruyama N, Kamano T, Kamiya Y, Fujita H, Nakagawa Y, Nagasaka M, Iwata M, et al. Increased number of methylated CpG islands correlates with Helicobacter pylori infection, histological and serological severity of chronic gastritis. Eur J Gastroenterol Hepatol. 2009; 21:613–19. 10.1097/MEG.0b013e32830e28b2. 19307977

[14] Tahara T, Shibata T, Nakamura M, Yamashita H, Yoshioka D, Okubo M, Yonemura J, Maeda Y, Maruyama N, Kamano T, Kamiya Y, Fujita H, Nakagawa Y, et al. Increased number of CpG island hypermethylation in tumor suppressor genes of non-neoplastic gastric mucosa correlates with higher risk of gastric cancer. Digestion. 2010; 82:27–36. 10.1159/000252766. 20150736

[15] To KF, Leung WK, Lee TL, Yu J, Tong JH, Chan MW, Ng EK, Chung SC, Sung JJ. Promoter hypermethylation of tumor-related genes in gastric intestinal metaplasia of patients with and without gastric cancer. Int J Cancer. 2002; 102:623–28. 10.1002/ijc.10783. 12448005

[16] Kang GH, Lee HJ, Hwang KS, Lee S, Kim JH, Kim JS. Aberrant CpG island hypermethylation of chronic gastritis, in relation to aging, gender, intestinal metaplasia, and chronic inflammation. Am J Pathol. 2003; 163:1551–56. 10.1016/S0002-9440(10)63511-0. 14507661 PMC1868290

[17] Kang GH, Lee S, Kim JS, Jung HY. Profile of aberrant CpG island methylation along the multistep pathway of gastric carcinogenesis. Lab Invest. 2003; 83:635–41. 10.1097/01.LAB.0000067481.08984.3F. 12746473

[18] Lee JH, Park SJ, Abraham SC, Seo JS, Nam JH, Choi C, Juhng SW, Rashid A, Hamilton SR, Wu TT. Frequent CpG island methylation in precursor lesions and early gastric adenocarcinomas. Oncogene. 2004; 23:4646–54. 10.1038/sj.onc.1207588. 15064707

[19] Sepulveda AR, Yao Y, Yan W, Park DI, Kim JJ, Gooding W, Abudayyeh S, Graham DY. CpG methylation and reduced expression of O6-methylguanine DNA methyltransferase is associated with Helicobacter pylori infection. Gastroenterology. 2010; 138:1836–44. 10.1053/j.gastro.2009.12.042. 20044995

[20] Waki T, Tamura G, Sato M, Terashima M, Nishizuka S, Motoyama T. Promoter methylation status of DAP-kinase and RUNX3 genes in neoplastic and non-neoplastic gastric epithelia. Cancer Sci. 2003; 94:360–64. 10.1111/j.1349-7006.2003.tb01447.x. 12824905 PMC11160204

[21] Sepulveda JL, Gutierrez-Pajares JL, Luna A, Yao Y, Tobias JW, Thomas S, Woo Y, Giorgi F, Komissarova EV, Califano A, Wang TC, Sepulveda AR. High-definition CpG methylation of novel genes in gastric carcinogenesis identified by next-generation sequencing. Mod Pathol. 2016; 29:182–93. 10.1038/modpathol.2015.144. 26769141

[22] Truong AH, Ben-David Y. The role of Fli-1 in normal cell function and malignant transformation. Oncogene. 2000; 19:6482–89. 10.1038/sj.onc.1204042. 11175364

[23] Vo KK, Jarocha DJ, Lyde RB, Hayes V, Thom CS, Sullivan SK, French DL, Poncz M. FLI1 level during megakaryopoiesis affects thrombopoiesis and platelet biology. Blood. 2017; 129:3486–94. 10.1182/blood-2017-02-770958. 28432223 PMC5492092

[24] Sakurai T, Kondoh N, Arai M, Hamada J, Yamada T, Kihara-Negishi F, Izawa T, Ohno H, Yamamoto M, Oikawa T. Functional roles of Fli-1, a member of the Ets family of transcription factors, in human breast malignancy. Cancer Sci. 2007; 98:1775–84. 10.1111/j.1349-7006.2007.00598.x. 17727680

[25] Song W, Li W, Li L, Zhang S, Yan X, Wen X, Zhang X, Tian H, Li A, Hu JF, Cui J. Friend leukemia virus integration 1 activates the Rho GTPase pathway and is associated with metastasis in breast cancer. Oncotarget. 2015; 6:23764–75. 10.18632/oncotarget.4350. 26156017 PMC4695150

[26] Scheiber MN, Watson PM, Rumboldt T, Stanley C, Wilson RC, Findlay VJ, Anderson PE, Watson DK. FLI1 expression is correlated with breast cancer cellular growth, migration, and invasion and altered gene expression. Neoplasia. 2014; 16:801–13. 10.1016/j.neo.2014.08.007. 25379017 PMC4212256

[27] Lin PC, Lin JK, Lin CH, Lin HH, Yang SH, Jiang JK, Chen WS, Chou CC, Tsai SF, Chang SC. Clinical Relevance of Plasma DNA Methylation in Colorectal Cancer Patients Identified by Using a Genome-Wide High-Resolution Array. Ann Surg Oncol. 2015; 22:S1419–27. 10.1245/s10434-014-4277-2. 25472652

[28] Oster B, Thorsen K, Lamy P, Wojdacz TK, Hansen LL, Birkenkamp-Demtröder K, Sørensen KD, Laurberg S, Orntoft TF, Andersen CL. Identification and validation of highly frequent CpG island hypermethylation in colorectal adenomas and carcinomas. Int J Cancer. 2011; 129:2855–66. 10.1002/ijc.25951. 21400501

[29] McCarty KS Jr, Szabo E, Flowers JL, Cox EB, Leight GS, Miller L, Konrath J, Soper JT, Budwit DA, Creasman WT, et al. Use of a monoclonal anti-estrogen receptor antibody in the immunohistochemical evaluation of human tumors. Cancer Res. 1986; 46:4244s–48s. 3524805

[30] Klijn C, Durinck S, Stawiski EW, Haverty PM, Jiang Z, Liu H, Degenhardt J, Mayba O, Gnad F, Liu J, Pau G, Reeder J, Cao Y, et al. A comprehensive transcriptional portrait of human cancer cell lines. Nat Biotechnol. 2015; 33:306–12. 10.1038/nbt.3080. 25485619

[31] Matsuda M, Watanabe A, Sawada H, Yamada Y, Nakano H, Iwai M, Iwai Y. Establishment of an alpha-fetoprotein-producing cell line derived from gastric cancer. In Vitro Cell Dev Biol Anim. 1999; 35:555–57. 10.1007/s11626-999-0090-9. 10614861

[32] Riggi N, Knoechel B, Gillespie SM, Rheinbay E, Boulay G, Suvà ML, Rossetti NE, Boonseng WE, Oksuz O, Cook EB, Formey A, Patel A, Gymrek M, et al. EWS-FLI1 utilizes divergent chromatin remodeling mechanisms to directly activate or repress enhancer elements in Ewing sarcoma. Cancer Cell. 2014; 26:668–81. 10.1016/j.ccell.2014.10.004. 25453903 PMC4492343

[33] Hart A, Melet F, Grossfeld P, Chien K, Jones C, Tunnacliffe A, Favier R, Bernstein A. Fli-1 is required for murine vascular and megakaryocytic development and is hemizygously deleted in patients with thrombocytopenia. Immunity. 2000; 13:167–77. 10.1016/S1074-7613(00)00017-0. 10981960

[34] Raslova H, Komura E, Le Couédic JP, Larbret F, Debili N, Feunteun J, Danos O, Albagli O, Vainchenker W, Favier R. FLI1 monoallelic expression combined with its hemizygous loss underlies Paris-Trousseau/Jacobsen thrombopenia. J Clin Invest. 2004; 114:77–84. 10.1172/JCI21197. 15232614 PMC437972

[35] Human Protein Atlas. FLI1. Available from v17.proteinatlas.org: https://www.proteinatlas.org/ENSG00000151702-FLI1/tissue. Accessed 13/10/2017.

[36] Shintani S, Hamakawa H, Nakashiro K, Shirota T, Hatori M, Tanaka M, Kuroshita Y, Kurokawa Y. Friend leukaemia insertion (Fli)-1 is a prediction marker candidate for radiotherapy resistant oral squamous cell carcinoma. Int J Oral Maxillofac Surg. 2010; 39:1115–19. 10.1016/j.ijom.2010.02.027. 20709497

[37] Guerrero-Preston R, Michailidi C, Marchionni L, Pickering CR, Frederick MJ, Myers JN, Yegnasubramanian S, Hadar T, Noordhuis MG, Zizkova V, Fertig E, Agrawal N, Westra W, et al. Key tumor suppressor genes inactivated by “greater promoter” methylation and somatic mutations in head and neck cancer. Epigenetics. 2014; 9:1031–46. 10.4161/epi.29025. 24786473 PMC4143405

[38] Bai G, Wu C, Gao Y, Shu G. Exploring the Functional Disorder and Corresponding Key Transcription Factors in Intraductal Papillary Mucinous Neoplasms Progression. Int J Genomics. 2015; 2015:197603. 10.1155/2015/197603. 26425543 PMC4573622

[39] Mhawech-Fauceglia P, Herrmann FR, Bshara W, Odunsi K, Terracciano L, Sauter G, Cheney RT, Groth J, Penetrante R, Mhawech-Fauceglia P. Friend leukaemia integration-1 expression in malignant and benign tumours: a multiple tumour tissue microarray analysis using polyclonal antibody. J Clin Pathol. 2007; 60:694–700. 10.1136/jcp.2006.039230. 16917000 PMC1955051

